# F16. GLUTAMATE AND GABA LEVELS IN ANTIPSYCHOTIC-NAïVE SCHIZOPHRENIA PATIENTS ARE ASSOCIATED WITH TREATMENT OUTCOME AFTER 1.5 AND 6 MONTHS

**DOI:** 10.1093/schbul/sby017.547

**Published:** 2018-04-01

**Authors:** Kirsten Bojesen, Brian Broberg, Kasper Jessen, Anne Sigvard, Karen Tangmose, Mette Ødegaard Nielsen, Egill Rostrup, Birte Glenthoj

**Affiliations:** 1 Center for Neuropsychiatric Schizophrenia Research (CNSR) and Center for Clinical Intervention and Neuropsychiatric Schizophrenia Research (CINS), Copenhagen University Hospital; 2 Center for Neuropsychiatric Schizophrenia Research; 3 Center for Neuropsychiatric Schizophrenia Research, and Center for Clinical Intervention and Neuropsychiatric Schizophrenia Research, University of Copenhagen; 4 CNSR, Copenhagen University Hospital; 5 Center for Neuropsychiatric Schizophrenia Research, and Center for Clinical Intervention and Neuropsychiatric Schizophrenia Research, University of Copenhagen, Glostrup University Hospital

## Abstract

**Background:**

Higher glutamate levels are found in the anterior cingulate cortex (ACC) of non-responder (NR) patients with schizophrenia in cross-sectional studies. However, it remains unclear if this reflects the pathophysiology of NR patients or the effect of antipsychotics and illness chronicity. Also, no previous study has assessed if levels of GABA in the ACC and glutamate in the thalamus are abnormal in NR patients from illness onset. To investigate this, we examined antipsychotic-naïve schizophrenia (SCZ) patients before and after treatment.

**Methods:**

Longitudinal study of 38 initially antipsychotic naïve SCZ patients and 34 matched healthy controls (HC) assessed at baseline, after 1.5 months (NSCZ=29, NHC=33), and 6 months (NSCZ=26, NHC=28) of treatment. Patients were treated with aripiprazole for the first 1.5 months (open label). Hereafter, treatment could be modified. Responders (R) and non-responders (NR) were assessed using the Andreasen criteria. Glutamate spectra in the ACC and left thalamus were acquired with a PRESS sequence, and GABA spectra in the ACC with a MEGA-PRESS on a 3T MR scanner.

**Results:**

First, the trajectory of glutamate/Cr and GABA/Cr levels were evaluated in SCZ patients and HC with a linear mixed model. In the left thalamus, a significant time*group interaction was observed (p=0.01) due to higher levels of glutamate/Cr in SCZ patients at baseline (p=0.03), but not after 1.5 and 6 months’ treatment as compared with HC. In the ACC, a significant main effect of group was found for both glutamate/Cr (p=0.04) and GABA/Cr (p=0.003) due to lower levels in SCZ patients at all examinations, and the time*group interactions were non-significant.

Secondly, we investigated if baseline levels of glutamate/Cr and GABA/Cr differed in NR patients after 1.5 and 6 months’ treatment using ANOVA. In the left thalamus, NR patients after both 1.5 and 6 months had significantly higher baseline glutamate/Cr compared with HC (P1.5months=0.03 and P6months<0.05), whereas R and HC did not differ. In the ACC, NR after 1.5 months showed a trend for lower GABA/Cr at baseline (p=0.06), and in NR after 6 months baseline GABA/Cr was significantly lower compared with HC (p=0.03), whereas R and HC did not differ. In the ACC, there was no baseline difference in glutamate/Cr of NR patients after 1.5 and 6 months compared with HC.

**Discussion:**

The findings indicate increased glutamatergic turnover in the left thalamus and decreased GABAergic neurotransmission in the ACC in the pathophysiology of schizophrenia. Treatment normalises glutamate levels in the left thalamus, but does not affect GABA and glutamate levels in the ACC. Importantly, NR patients are characterized by more pronounced glutamatergic and GABAergic disturbances in the antipsychotic-naïve state. Compounds that modify glutamatergic and GABAergic neurotransmission might have therapeutic potential in this subgroup.

